# TRPC Channels as Mediators of Hypoxia-Induced Pulmonary Hypertension in Obstructive Sleep Apnea

**DOI:** 10.3390/ijms27041861

**Published:** 2026-02-15

**Authors:** Yolima P. Torres, Andrés Felipe Aristizábal-Pachón, Liliana Otero

**Affiliations:** 1 Departamento de Nutrición y Bioquímica, Facultad de Ciencias, Pontificia Universidad Javeriana, Bogotá 110231, Colombia; andres_aristizabal@javeriana.edu.co; 2 Centro de Investigaciones Odontológicas (CIO), Faculty of Dentistry, Pontificia Universidad Javeriana, Bogotá 110311, Colombia; lotero@javeriana.edu.co

**Keywords:** TRPC channels, obstructive sleep apnea, pulmonary hypertension, hypoxia

## Abstract

Pulmonary hypertension (PH) is a progressive disorder characterized by elevated pulmonary arterial pressure and the extensive remodeling of pulmonary vasculature. Chronic intermittent hypoxia (CIH), a hallmark of obstructive sleep apnea (OSA), is a well-established contributor to the pathogenesis of PH. OSA is defined by repetitive episodes of upper airway obstruction during sleep, leading to cycles of hypoxia and reoxygenation that trigger a cascade of deleterious events including oxidative stress, inflammation, endothelial dysfunction, and vascular remodeling. Growing evidence underscores the critical role of transient receptor potential canonical (TRPC) channels in mediating hypoxia-induced vascular alterations that contribute to the development of PH. TRPC channels are non-selective cation channels that regulate calcium influx in response to mechanical stimuli, pro-inflammatory cytokines, oxidative stress, and hypoxia. These channels are expressed in both pulmonary arterial smooth muscle cells (PASMCs) and pulmonary artery endothelial cells (PAECs), where they modulate key processes such as proliferation, migration, apoptosis, endothelial permeability, and vasoconstriction. Under hypoxic conditions, the upregulation of TRPC1, TRPC3, TRPC4, and TRPC6 has been implicated in dysregulation of calcium homeostasis and activation of pathological signaling pathways that contribute to increased pulmonary arterial pressure. In this review, we propose that upregulation and functional modulation of TRPC channels under CIH represents a central pathogenic mechanism linking OSA to PH. We hypothesize that TRPC1, TRPC3, TRPC4, and TRPC6 act as critical molecular effectors mediating hypoxia-driven calcium influx and downstream signaling pathways that lead to pulmonary vascular remodeling, endothelial dysfunction, and increased pulmonary arterial pressure. This framework allows us to integrate mechanistic insights from molecular, cellular, and translational studies, and to evaluate the therapeutic potential of targeting TRPC channels in OSA-associated PH.

## 1. Introduction

Pulmonary hypertension (PH) is a progressive, severe condition characterized by elevated pulmonary arterial pressure and the significant structural remodeling of the pulmonary vasculature. Among its multiple etiologies, chronic exposure to hypoxia is a well-recognized driver of pathogenesis, particularly in conditions such as OSA [1,2]. OSA is one of the most prevalent sleep-related breathing disorders, affecting nearly one billion individuals globally. It is defined by repetitive episodes of upper airway obstruction during sleep, leading to CIH, a critical pathophysiological insult that initiates a cascade of adverse events, including oxidative stress, systemic and pulmonary inflammation, endothelial dysfunction, and pulmonary vascular remodeling, all of which contribute to the development and progression of PH [3,4].

Among the diverse molecular mechanisms implicated in PH, the dysregulation of ion channel activity plays a central role. TRPC channels have been recognized as key modulators of vascular physiology and pathophysiology [5]. TRPC channels are non-selective cation channels that mediate calcium and sodium influx in response to diverse stimuli, including mechanical stretch, pro-inflammatory cytokines, growth factors, oxidative stress, and hypoxia [6]. Their expression in both PAECs and PASMCs allows them to regulate critical processes including cell proliferation, migration, apoptosis, and vasoconstriction [7]. TRPC channels act either as receptor-operated calcium channels (ROCCs) or store-operated calcium channels (SOCCs), positioning them as essential components of calcium homeostasis [8].

Hypoxia significantly alters the expression and activity of specific TRPC isoforms, including TRPC1, TRPC3, TRPC4 and TRPC6 [9]. Their upregulation has been associated with increased calcium entry and activation of downstream signaling pathways, and PASMCs proliferation leading to arterial wall muscularization [10]. Significantly, TRPC subunits form homo- or hetero-tetrameric complexes, enhancing their regulatory versatility in pulmonary vascular responses to hypoxia. Experimental models of CIH and chronic hypoxia (CH) consistently demonstrated upregulation of TRPC3 and TRPC6, correlating with increased calcium influx, oxidative stress, and pathological pulmonary vascular remodeling [11,12]. Pharmacological blockade or genetic silencing of these channels in animal models has been shown to attenuate the development of PH and improve hemodynamic parameters in preclinical models, supporting their therapeutic potential [13]. Similarly, TRPC1 and TRPC4 have been implicated in PASMCs migration and EC apoptosis, suggesting that their dysregulation contributes to structural and functional vascular alterations in OSA [14,15].

Taken together, these findings suggest that TRPC channels are central regulators of vascular pulmonary tone and remodeling under hypoxic conditions. The dual expression in PAECs and PASMCs highlights the opportunity for therapeutic interventions targeting multiple levels of vascular dysfunction. In this context, this review proposes that CIH, as occurs in OSA, leads to sustained activation and upregulation of TRPC channels in pulmonary vascular cells, thereby amplifying Ca^2+^ influx and triggering proliferative, contractile, and inflammatory pathways that drive pulmonary vascular remodeling and hypertension. The mechanistic context proposes that TRPC-mediated calcium signaling acts as a unifying mechanism linking hypoxia, endothelial dysfunction and smooth muscle hyperreactivity, offering and integrated explanation for the vascular pathology observed in OSA-associated PH. Accordingly, this review integrates experimental and preclinical evidence to clarify the molecular mechanisms by with TRPC channels contribute to pulmonary dysfunction and to identify these channels as potential therapeutic targets for future intervention. To better understand these processes, it is necessary to establish the molecular and cellular basis of PH. This involves the complex interplay between endothelial and smooth muscle, calcium-dependent signaling, and the regulatory networks that maintain vascular tone. The following section explores these fundamental mechanisms, setting the stage for subsequent discussion of how TRPC channel activation integrates into hypoxia-driven vascular remodeling.

## 2. Molecular Mechanisms Involved in Pulmonary Hypertension

Pulmonary hypertension is a complex, multifactorial disease clinically defined by a resting mean pulmonary arterial pressure (mPAP) exceeding 20 mmHg and characterized by dysfunctions of pulmonary arterial cells and sustained elevation in pulmonary pressure, primarily driven by persistent vasoconstriction [1]. In addition, there is a progressive pulmonary vascular remodeling of small pulmonary arteries, involving enhanced cell proliferation, and resistance to apoptosis [16,17,18,19]. The pulmonary arterial wall comprises endothelial cells (ECs) and vascular smooth muscle cells (VSMCs), which work in close coordination to regulate vascular function and are central in the pathophysiology of PH [20,21].

Under physiological conditions, ECs modulate vascular tone and homeostasis via the balanced release of vasoactive mediators such as nitric oxide (NO) and endothelin-1 (ET-1), which function as a vasodilator and vasoconstrictor, respectively. In pathological conditions, ECs can adopt pro-inflammatory phenotypes and release factors promoting smooth muscle dysfunction [20,22]. VSMCs exhibit phenotypic plasticity, switching from a differentiated and contractile state to a dedifferentiated and proliferative state in response to vascular injury or pathological stimuli such as hypoxia or inflammation. In the differentiated state, VSMCs express contractile proteins, ion channels, and signaling molecules necessary for regulating vascular tone and flow. When dedifferentiated, these cells re-enter the cell cycle, promoting pathological changes like increased proliferation, migration, fibrosis, and inflammation in the vascular wall [23,24]. The interaction between VSMCs and ECs is essential for managing blood pressure and disruption of EC-VSMCs crosstalk exacerbates vasoconstriction and remodeling, central hallmarks of PH [20,22].

Precise control of intracellular calcium concentration ([Ca^2+^]_i_) is essential for vascular cell function [17,25]. In ECs, Ca^2+^ modulates membrane potential, NO synthesis, permeability, and proliferative signaling [26]. In VSMCs, calcium homeostasis plays a pivotal role in regulating contractile function through a well-organized cytoskeletal network. Transient rises in [Ca^2+^]_i_ activate myosin light chain kinase (MLCK) via calcium—calmodulin binding, leading to phosphorylation of myosin light chain and contraction. Relaxation occurs when [Ca^2+^]_i_ decrease by efflux or uptake into the endoplasmic reticulum (ER)/sarcoplasmic reticulum (SR), activating myosin light chain phosphatase (MLCP). Dysregulated calcium homeostasis thus drives sustained vasoconstriction, VSMCs hypercontractility, growth and matrix remodeling [23]. Two main pathways mediate [Ca^2+^]_i_ entry into VSMCs: Voltage-dependent calcium channels (VDCC), especially L-type channels activated upon membrane depolarization, and non-voltage gated Ca^2+^ channels, including receptor-operated Ca^2+^ entry (ROCE) and store-operated Ca^2+^ entry (SOCE), which are linked primarily to receptor and ER/SR signaling rather than electrical stimuli [27,28,29,30]. SOCE is triggered when inositol 1,4,5-triphosphate (IP3) stimulates Ca^2+^ release from the ER/SR via IP3 receptors (IP3). The resulting depletion of ER stores is sensed by stromal interaction molecule (STIM) proteins (mainly STIM1), which relocate and activate plasma membrane channels, classically Orai and non-selective TRPC channels generating sustained Ca^2+^ influx. This STIM-Orai-TRPC axis is central to maintaining Ca^2+^ homeostasis and translating store depletion into downstream signaling [30,31,32,33,34].

Accumulating evidence implicates TRPC (Transient receptor potential canonical) channels, especially TRPC1, TRPC3, TRPC4 and TRPC6, in pathological Ca^2+^ influx contributing to PH pathophysiology [35,36,37,38]. In addition, pathologic conditions such as hypoxia induce modifications of TRPC expression and activity [39]. In the following sections, we explore the structural properties, modulatory pathways, and pathophysiological contributions of TRPC channels in pulmonary vascular physiology, with particular attention to their role in hypoxia-driven PH. Overall, these observations emphasize that calcium signaling serves as a key regulator of pulmonary vascular tone and remodeling. Within this context, TRPC channels emerge as central molecular effectors translating hypoxic and mechanical cues into calcium-dependent vascular responses. The next section examines their structural characteristics, activation mechanisms, and specific contributions to PH.

## 3. TRPC Channels in Pulmonary Vascular Physiology and Pathophysiology

### 3.1. TRPC Channel’s Structure and Modulation

Transient receptor potential canonical (TRPC) channels are non-selective cation channels that mediate Ca^2+^ and Na^+^ flux in response to diverse chemical and mechanical stimuli. TRPC channels consist of six transmembrane segments with the pore domain located between the S5 and S6 segments. The intracellular N- and C- terminal regions are critical for channel regulation and interactions with other cellular proteins [40,41,42,43]. Similarly to other members of the TRP family, the N-terminal region of TRPC channels contain ankyrin repeat domains, which are essential for protein–protein interaction. The C-terminal region includes a Calmodulin- and IP3 receptors-binding (CIRB) site, which interacts with various regulatory molecules, such as phosphoinositides, inositol polyphosphates and Gα_i/o_ proteins, to modulate channel activity [43,44].

There are seven members of TRPC channels (TRPC1-TRPC7) which are classified into four subgroups according to the amino-acid sequence: TRPC1, TRPC2, TRPC3/6/7, and TRPC4/5. They can assemble into homo- or heterotetramers which enhances their functional and regulatory diversity across different cell types and tissues (Table 1).

The regulatory mechanisms governing these channels are complex and involve multiple signaling pathways, lipid mediators, and protein–protein interaction, with phospholipase C (PLC) signaling serving as a key activation mechanism (Table 1). Upon activation of the Gα_q/11_- coupled receptor, PLC hydrolyzes phosphatidylinositol 4,5 biphosphate (PIP2) to produce the second messenger diacylglycerol (DAG) and IP_3_ [45,46]. DAG activates diverse TRPC channels including TRPC3, TRPC6, and TRPC7 [40,43]. Meanwhile, IP3 acts through IP3 receptors to activate TRPC1, TRPC3, and TRPC4 [47,48]. PIP2 modulates both upregulation and downregulation of different TRPC members [49,50]. Intracellular Ca^2+^ exerts a dual regulatory effect on TRPC channels, acting through direct binding as well as via Ca^2+^ -calmodulin (CaM) and CAM-dependent kinase II (CAMKII) proteins [51]. Protein kinases A and C can regulate these channels through phosphorylation of specific residues on TRPC3 and TRPC5 [52,53]. Additionally, TRPC1, TRPC5 and TRPC6 are activated by mechanosensitive stimuli, including hypoosmotic conditions and membrane stretch [54,55]. Oxidative stress has also been identified as a modulator of TRPC channel activity through redox modification of the free thiol groups. Reactive oxygen species (ROS) directly activate TRPC4 and TRPC5 via redox-sensitive N-terminal cysteine residues C549/C554 and C553/C558, respectively. Moreover, the TRPC6 channel can be indirectly activated through extracellular signal regulated kinase (ERK) pathway in response to oxidative stress [56,57]. Given their broad expression and the ability to integrate a wide range of physiological and pathological stimuli, TRPC channels have emerged as critical regulators in cardiovascular pathophysiology.

**Table 1:** Activation mechanisms and tetrameric assembly types of TRPC channels. This table summarizes the primary activation mechanisms and subunit assembly configurations of TRPC channels relevant to pulmonary vascular physiology and hypoxia-induced pulmonary hypertension, including the known tetrameric assemblies of TRPC channels. Abbreviations: PAECs, pulmonary arterial endothelial cells; PASMCs, pulmonary arterial smooth muscle cells; IP3, Inositol triphosphate; PIP2, phosphatitylinositol 4,5 biphosphate; DAG, Diacylglycerol, PLC, Phospholipase C; PAH, pulmonary arterial hypertension; eNOS, Endothelial NO synthase; NO, nitric oxide, CIH, chronic intermittent hypoxia.

Channel	Activation Mechanism	Tetrameric Assembly Type
TRPC1	IP3-, PIP2- and oxidative stress-sensitive [47,48,49,56]	Heterotetrameric (TRPC1/TRPC4; TRPC1/TRPC5; TRPC1/TRPC6) [58,59,60]
TRPC3	DAG-and IP3-sensitive, PIP2-modulated [48,61,62,63]	Homo- and heterotetrameric (TRPC3/TRPC6; TRPC3/TRPC7) [59,64]
TRPC4	G protein/PLC—Ca^2+^/PIP2-dependent [65,66]	Homo- and heterotetrameric (TRPC1/TRPC4) [64]
TRPC5	PIP2-and oxidative stress-sensitive [57,67,68]	Homo- and heterotetrameric (TRPC1/TRPC5) [60,64]
TRPC6	DAG- and oxidative stress-sensitive [56,63]	Homo- and heterotetrameric (TRPC1/TRPC6; TRPC3/TRPC6) [59]
TRPC7	DAG-sensitive [64]	Homo- and heterotetrameric (TRPC3/TRPC7) [64]

### 3.2. The Role of TRPC Channels in Pulmonary Hypertension

TRPC channels are widely expressed in vascular cells, where they play a role in controlling vascular tone and are involved in the onset of vascular conditions, such as systemic and PH [5,43,69]. TRPC1, TRPC3, TRPC4, TRPC5, and TRPC6 channels are found in both endothelial and smooth muscle cells, where they integrate chemical, mechanical and ionic signals to regulate intracellular calcium levels [7,8,70,71,72,73]. The activation of TRPC channels in EC is linked to several endothelial functions, including the regulation of intracellular calcium, which subsequently influence the production of vasoactive molecules. TRPC1 and TRPC4 facilitate vasodilation by activating endothelial NO synthase (eNOS) and releasing NO [8,74,75,76] while TRPC3 is involved in hyperpolarization-mediated vasodilation [77]. In addition, TRPC5 mediates the increase in production of ROS [5,69].

In VSMCs, TRPC1, TRPC3, TRPC4, TRPC5, and TRPC6 contribute to calcium influx, and alterations in their expression can affect vascular tone, promoting cell proliferation and pulmonary vascular remodeling (Table 2) [39,70,78,79,80,81,82,83]. In PASMCs, these channels are responsible for SOCE, mediating proliferation and resistance to apoptosis. Furthermore, TRPC1 and TRPC4 participate in migration capacity [39], while TRPC3 and TRPC6 regulate vascular tone by promoting vasoconstriction [78]. Changes in TRPC expression and activity have been associated with PH. In PASMCs dysfunction in pulmonary arterial hypertension (PAH), TRPC3 and TRPC6 are overexpressed, correlating with pathological vascular changes. By contrast, TRPC4 is reduced, suggesting a shift in TRPC channels expression in the disease state and selective upregulation during disease progression [39].

Compensatory changes in TRPC expression have been observed in various pathological conditions. For example, in VSMCs TRPC6−/− mice, TRPC3 is upregulated to compensate for the loss of TRPC6 activity [85]. Aging vessels exhibit a similar pattern, with increased TRPC6 expression and decreased TRPC1 levels, impairing endothelium-dependent vasorelaxation and contributing to age-related vasospastic disorders [86]. In PH induced by hypoxia, there are changes in TRPC6 and TRPC1 expression without changes in calcium signaling as TRPC6 is upregulated while TRPC1 is downregulated, suggesting a compensatory mechanism [87]. Similarly, when TRPC1 was silenced, TRPC6 expression was enhanced, and ROCCs activity was not affected [88]. Conversely, TRPC1 overexpression in hyperplastic smooth muscle cells is associated with enhanced calcium entry and proliferation, which can be mitigated by TRPC1 inhibitor [81]. In a model of essential hypertension increased expression of TRPC3 and TRPC6 was observed, suggesting a higher proportion of TRPC3 homotetramers and TRPC3/TRPC6 complexes with an elevated TRPC3 subunit ratio [84].

TRPC channels are crucial regulators of vascular tone and structure in both endothelial and smooth muscle cells. Their involvement in calcium signaling pathways central to cardiovascular homeostasis and disease makes them promising therapeutic targets in conditions such as PH. Pharmacological modulation of these channels demonstrated therapeutic potential. For instance, the vasodilator chloroquine (CLQ) downregulates both TRPC1 and TRPC6 expression, thereby inhibiting calcium entry through SOCCs and ROCCs [89,90]. TRPC3 has also been identified as a promising therapeutic target for treating PH in PASMCs because of its role in proliferation, migration, and apoptosis resistance [39].

While TRPC channels play a vital role in maintaining normal vascular function, their relevance becomes heightened under pathological conditions such as PH. In particular, OSA, characterized by recurrent episodes of CIH, has been consistently associated with the development and progression of PH, creating a conducive environment for the TRPC channel dysfunction. Collectively, these findings establish a mechanistic foundation connecting TRPC dysregulation to hypoxia-driven vascular remodeling. This relationship gains relevance in OSA, where recurrent hypoxic episodes activate similar calcium-dependent pathways contributing to PH.

## 4. Obstructive Sleep Apnea and Its Association with Pulmonary Hypertension

OSA is a sleep breathing disorder, characterized by repeated episodes of upper airway collapse leading to intermittent hypoxia, sleep fragmentation, and systemic inflammation. It is recognized as a global health issue, affecting nearly one billion individuals worldwide, with prevalence increasing with age and obesity [3,91]. OSA is strongly associated with cardiovascular diseases, including systemic hypertension, coronary artery disease, heart failure, and arrhythmia, via mechanisms such as oxidative stress, endothelial dysfunction, and heightened sympathetic activity [4,92].

Although OSA is not classified as a direct cause of PH [1], numerous studies demonstrate that it contributes to the development of mild to moderate PH, often as a comorbidity, with prevalence influenced by age, obesity, and coexisting chronic obstructive pulmonary disease (COPD) [2,93,94,95,96,97]. Moreover, the coexistence of OSA and COPD further increases the risk of developing PH, which correlates with the severity of OSA [98]. OSA contributes to PH through a combination of hemodynamic, structural, and biochemical mechanisms such as intermittent hypoxia, sympathetic nervous system activation, endothelial dysfunction and vasoactive mediators, left heart dysfunction, mechanical and hemodynamic factors, inflammation and oxidative stress [99,100].

There is a bidirectional relationship between OSA and PH, with approximately 25% patients with PH presenting concomitant OSA [101]. The estimated prevalence and severity of OSA vary across studies due to differences in study populations, sample sizes, and the apnea–hypopnea index (AHI) thresholds used [102]. In clinical cohorts, the prevalence of PH in OSA ranges from 15% to 65%, with higher rates observed in severe OSA and in women [103,104,105,106]. In pediatric OSA, PH is less frequent (1.8% to 8%), but is strongly associated with comorbidities, with up to 90% of children with PH also having OSA [107,108,109,110]. Therapeutically, continuous positive airway pressure (CPAP) remains the gold standard for managing OSA. CPAP has been shown to reduce pulmonary pressures, improve endothelial function, and decrease oxidative stress in OSA patients with PH [15,94,111]. While generally safe and effective, CPAP responses can vary among patients, and some studies have reported transient increased levels of endothelial injury biomarkers such as angiopoietin-2 [112,113]. However, the clinical significance of these findings remains uncertain and has not been linked to adverse outcomes. There remains a need for longitudinal and interventional clinical studies to clarify the causal relationship between OSA and PH, including the potential reversibility of pulmonary vascular changes with CPAP.

At molecular level, CIH in OSA along with hypercapnia leads to acute pulmonary vasoconstriction and increased pulmonary artery pressure [100]. One key process is hypoxic pulmonary vasoconstriction (HPV), which induces proliferation of VSMCs and pathological pulmonary vascular remodeling [4,114]. Hypoxia also promotes proliferation of the tunica intima, resulting in occlusion of distal pulmonary arteries and impaired pulmonary blood flow [2]. CIH also disrupts the balance of vasoactive mediators, increasing ET-1 and reducing NO and prostacyclin, molecules essential for the regulation of vasoconstriction and pulmonary vascular remodeling [115,116]. Systemic and local inflammation also play central roles. Elevated levels of pro-inflammatory cytokines such as Interleukin-6 (IL-6) and tumor necrosis factor (TNF-α), adhesion molecules including intercellular adhesion molecule (ICAM) and vascular cell adhesion molecule (VCAM) as well as markers like C-reactive protein (CRP), have been consistently observed in OSA and contribute to pulmonary vascular remodeling [2,4,96,117]. Moreover, CIH stabilizes hypoxia-inducible factors (HIF-1α), which regulate genes promoting angiogenesis, proliferation, and apoptosis resistance, further enhancing pulmonary vascular remodeling [118,119].

Finally, dysregulation of calcium homeostasis represents another critical mechanism: CIH alters calcium signaling in PASMCs and PAECs, leading to sustained increases in intracellular calcium concentration and activation of nuclear factor of activated T-cells (NFAT)-dependent transcriptional pathways, thereby amplifying vasoconstriction and pulmonary vascular remodeling [120]. Together, these mechanisms highlight the complex and multifactorial nature of OSA-induced PH, in which hypoxia, inflammation, and vascular dysfunction converge to drive disease progression. Despite significant preclinical advances, most mechanistic data originates from animal models that cannot replicate the full clinical heterogeneity of OSA, particularly in the presence of comorbidities such as obesity, COPD, or metabolic disorders. This gap underscores the need for well-designed clinical trials and mechanistic human studies to validate experimental findings and define causal pathways. TRPC channels, in particular are central regulators of calcium influx and vascular reactivity under hypoxia conditions. Their involvement in OSA-associated PH requires further research, as they may represent promising therapeutic targets for preventing or attenuating PH progression in this context. Accordingly, the following chapter examines the contribution of TRPC channels to pathological mechanisms underlying hypoxia-derived PH. The CIH observed in OSA shares key molecular features with experimental models of sustained hypoxia, including altered calcium homeostasis, oxidative stress, and upregulation of TRPC channel activity. These convergent mechanisms form the conceptual foundation for exploring how TRPC channels contribute to PH driven by hypoxic stress, thereby bridging experimental and clinical perspectives on calcium-dependent vascular remodeling.

## 5. Role of TRPC Channels in Progression of Hypoxia-Derived Pulmonary Hypertension

Preclinical models, including both animal studies and in vitro cellular approaches, have provided the bulk of evidence supporting the role of TRPC channels in PH induced by hypoxic stress, with diverse roles according to isoform. TRPC, Orai and STIM1 channels act as critical mediators of Ca^2+^ influx during HPV, thereby contributing to smooth muscle proliferation and pulmonary vascular remodeling during both acute and chronic hypoxic challenges. The role of TRPC1, TRPC4 and TRPC6 channels after acute hypoxic stimuli is related to a rapid Ca^2+^ influx that promotes PASMCs contraction, the hallmark of HPV. In contrast, sustained exposure to CH induces overexpression of TRPC1 and TRPC6, favoring abnormal cell proliferation, migration and pulmonary vascular remodeling, associated with the development of chronic PH [121]. Supporting this concept, Malczyk et al. (2013) [122] demonstrated that PASMCs exposed to CH (10% O_2_ for 21 days) selectively overexpressed TRPC1, while TRPC3 and TRPC6 remained unchanged. TRPC1 knockdown significantly reduced PASMCs proliferation, attenuated (Right ventricular systolic pressure) RVSP, and decreased muscularization of small pulmonary arteries, highlighting TRPC1 as key driver of pulmonary vascular remodeling [122]. Extending these findings, Sun et al. (2014) [11] showed in a murine model of hypoxia-induced PAH (11% O_2_ for 28 days) that silencing TRPC1 with siRNA significantly reduced RVSP and improves pulmonary vascular remodeling indices, including medial thickness and the number of muscularized vessels. Moreover, TRPC1 knockdown prevented the upregulation of TRPC1, TRPC4 and TRPC6, as well as HIF-1α, fibrotic markers such as TGF-β and p-Smad3, and pro-inflammatory mediators including TNF-α, matrix metalloproteinase-9 (MMP-9), and ET-1. Collectively, these results provided evidence that TRPC1 plays a central role in the pathogenesis of hypoxia-induced PAH and that its suppression can mitigate pulmonary vascular remodeling and attenuate disease progression [11]. The collective evidence provided by Malczyk et al. (2013) and Sun et al. (2014) [11,122] supported the implication of TRPC1 in pulmonary vascular remodeling induced by CH as the channel is selectively upregulated in PASMCs during sustained hypoxia. When the channel is silenced, medial thickening, RVSP and PASMCs proliferation are reduced [11,122].

Complementing this, Castillo-Galán et al. (2020) [9] provided direct experimental evidence in a rat model of CIH of a more complex pattern of TRPC function in hypoxic-derived PH. Their findings demonstrated that sustained exposure to CIH for 14, 21, and 28 days led to a progressive upregulation of TRPC1, TRPC4 and TRPC6 mRNA, with protein expression of TRPC4 and TRPC6 increasing as early as day 14 and continuing to rise at 21 and 28 days of hypoxic stimuli. These molecular changes were correlated with enhanced Ca^2+^ influx, higher RVSP and increase pulmonary vascular remodeling, thereby confirming a temporal association between TRPC upregulation and the progression of PH under CIH [9]. Extending this evidence, Castillo-Galán et al. (2022) [10] demonstrated that treatment with the non-selective TRPC blocker amynoethoxydiphenyl borate (2-APB), administered from day 14 to 28 of CIH exposure, prevented the increase in RVSP and reduced pulmonary vascular remodeling and PASMCs proliferation. These results provided functional confirmation that calcium influx through the TRP-STIM-Orai complex is critical for the development of PH under CIH. However, since 2-APB is a non-selective inhibitor, it does not permit determination of the specific role of isoform and the precise contribution of distinct TRPC channels [10].

Using genetic studies, Malkmus et al. (2022) [13] investigated the combined role of TRPC1, TRPC3 and TRPC6 in a murine model of CH (10% O_2_, 28 days) and provided further insight into isoform-specific functions. Triple knockout mice of TRPC1/3/6−/− abolished both the acute and sustained phases of HPV and significantly promoted a decrease in RVSP, pulmonary vascular resistance, and right ventricular wall thickness. Compared with wild-type controls, there were not observed differences in vascular muscularization, PASMCs proliferation, or migration. Instead, the protective effect was attributed to a near-complete loss of HPV. These findings suggest that TRPC1/3/6 are indispensable for the contractile component of HPV, but not necessarily for pulmonary vascular remodeling [13]. In contrast, using a murine model of CIH (5–21% O_2_ cycles for 4 weeks), mimicking the pathophysiological conditions of OSA, Park et al. (2023) [12] reported that TRCP3 deletion did not modify pulmonary vascular remodeling under CIH. However, channel silencing markedly worsens right ventricular dysfunction, increased wall thickness and upregulate stress markers, ROS production, and ET pathways. This differential phenotype highlights that TRPC3 plays a protective role in maintaining right ventricular resistance under hypoxic stress, diverging from the pathogenic role’s attributes to TRPC1, TRPC4, and TRPC6 in pulmonary vascular remodeling during hypoxia-induced PH [12].

Adding evidence of the TRPC role in ECs, using a mouse model of SY5416+ CH (HySu), rat model of monocrotaline-induced PH, and hypoxia-exposed PAECs, Yang et al. 2024 [123] demonstrate that TRPC4 is selectively upregulated in hypoxia-induced PAECs but not in PASMCs. Unlike TRPC1 and TRPC6, which act primarily in smooth muscle, TRPC4 promoted endothelial apoptosis via ER stress-caspase-12 signaling and upregulation of the proapoptotic protein Sudsd2, thereby contributing to pulmonary vascular remodeling and PH progression. Both shRNA-mediated knockdown and pharmacological inhibition with TRPC4 antagonist ML204 significantly attenuated RVSP, RV hypertrophy, ROS and apoptosis to values close to normoxic controls. These findings highlight TRPC4 as a critical endothelial mediator of hypoxia-driven PH and expand the repertoire of TRPC channels beyond smooth muscle proliferation and vasoconstriction and include significant changes in ECs [123].

Preclinical studies have been consistently demonstrated that Ca^2+^ influx via TRPC channels is indispensable for the pathogenesis of hypoxia-induced PH. These channels orchestrate key processes such as PASMCs proliferation, hypoxia-induces PH, or endothelial dysfunction, which collectively drive pulmonary vascular remodeling and disease progression [9,121]. Isoform-specific functions have also emerged. TRPC1 has been consistently implicated as a central regulator of PASMCs proliferation and structural remodeling [11,122], whereas TRPC3 appears to contribute to right ventricular adaptation under conditions of CIH highlighting a role that extends beyond pulmonary vasculature [9]. More recently, an endothelial perspective has been added, with TRPC4 identified as a key mediator of apoptosis through ER stress signaling, thereby linking TRPC signaling to endothelial dysfunction and pulmonary vascular remodeling [123].

Despite significant progress in elucidating TRPC channel-dependent mechanisms, preclinical evidence remains limited by marked variability in experimental conditions. Studies investigating hypoxia-induced PH have employed diverse experimental conditions, ranging from continuous hypoxia (10% O_2_ for 21–28 days) in rodents [13] to CIH (5–21% O_2_ cycles, 8h/day for 3–4 weeks) [9,10,12], or severe acute hypoxia (1% O_2_ for 30–120 min) in cellular assays [123]. Such differences in oxygen levels, exposure duration, and recovery intervals profoundly affect TRPC regulation, Ca^2+^ influx dynamics and vascular remodeling responses. In addition, pharmacological interventions show wide variability because the use of nonselective inhibitors such as 2-APB (50–100 μM) or more selective compounds such as ML204 (1–10 μM) or BI-749327 (1–3 mg/kg/day) and SH045 (3–10 mg/kg). Furthermore, the use of different experimental models, including rat and mouse PASMCs, PAECs, and in vivo models of CH or monocrotaline-induced PH, makes direct comparisons among studies difficult [10,123,124,125,126]. Future research should aim to standardize hypoxia exposure parameters and establish complete dose–response profiles using selective TRPC modulators, which is essential to strengthen the translational potential of TRPC-targeted therapies in hypoxia-induced PH.

Collectively, these findings establish TRPC channels as multifactorial contributors to hypoxia-driven PH, with roles that span smooth muscle contraction, proliferative remodeling, and endothelial injury. Nonetheless, gaps remain. Redundancy among TRPC isoforms complicates causal attribution, as compensatory upregulation of one isoform often occurs upon deletion of another [13]. Furthermore, the lack of isoform-selective pharmacological inhibitors limits the capacity to dissect specific molecular contributions [10]. These limitations underscore the need for integrative approaches combining genetic models and high-specific modulators, to resolve the functional roles within the TRPC family.

Translational studies in humans and clinical cohorts provide complementary support for the involvement of TRPC channels in human PAH, even though they are not always directly linked to hypoxia. Yu et al. (2004) demonstrated that TRPC3 and TRPC6 were markedly overexpressed in pulmonary tissues and primary PASMCs derived from patients with idiopathic pulmonary PH (IPAH), with TRPC6 knockdown significantly reducing this overexpression [127]. Extending these findings, Pousada et al. (2015) identified genetic variants in TRPC6 associated with susceptibility to PAH, which correlated with disease severity, underscoring a potential role of TRPC signaling in human pathology [128]. Although these findings were not conducted under hypoxic paradigms, they align with the preclinical evidence by confirming that TRPC overexpression and dysregulation are central features of the pulmonary vascular phenotype in PAH. Collectively, these lines of evidence establish TRPC channels as pivotal contributors to pulmonary vascular remodeling across experimental hypoxia-driven models and clinical PAH, while highlighting the need for translational studies that directly link hypoxia-related mechanisms and PH. Existing evidence positions TRPC channels as pivotal mediators of vascular remodeling in hypoxia-induced PH. In addition, translation of these understandings into effective therapies remains constrained by pharmacological limitations. These challenges are further addressed in the following section, which examines TRPC channels as emerging therapeutic targets.

## 6. TRPC Channels as Emerging Therapeutic Targets in Hypoxia Induced Pulmonary Hypertension

Experimental evidence from preclinical models has consistently demonstrated that TRPC1, TRPC3, TRPC4, and TRPC6 contribute to key pathogenic mechanisms of PH derived from hypoxia. However, the absence of isoform-selective pharmacological modulators for TRPC1 and TRPC3, along with the limited specificity of available TRPC4 antagonist, have constrained their translation into therapeutic strategies. In contrast, TRPC6 has attracted particular interest as several potent and relatively selective inhibitors have been developed and tested in PH models, yielding reproducible benefits on pulmonary pressure, remodeling and right ventricular adaptation (Table 3). Consequently, TRPC6 currently represents the most advanced pharmacological target within the TRPC family with multiple compounds acting as channel inhibitors. However, only a subset of these inhibitors has been evaluated directly in models of hypoxia-induced PH. Inhibition of TRPC6 with BI-749327 effectively reduced pulmonary vasoconstriction, right ventricular hypertrophy (RVH), and pulmonary vascular remodeling in hypoxia-induced PH by 40–50%. Furthermore, TRPC6 blockade suppressed Ca^2+^ influx and proliferative signaling pathways including protein kinase B (AKT) and mechanistic target of rapamycin (mTOR) phosphorylation in PASMCs, underscoring its dual action on vasoconstriction and remodeling [129]. Despite these promising findings, BI-749327 remains at the preclinical stage with no validation in human clinical trials. Additionally, although its activity is 85-fold more selective for TRPC6 over TRPC3, its selectivity is not absolute leaving open the possibility of no specific actions [124].

Another relevant compound is SAR7334, which has been characterized as a potent inhibitor of TRPC6 channels, with significantly higher selectivity for TRPC6 over TRPC isoforms. In experimental models of PH, treatment with SAR7334 effectively prevented rise in pulmonary arterial pressure induced by hypoxia, highlighting its therapeutic potential [130]. However, no clinical trials have been reported to date, and translational studies are still required to establish its safety and efficacy in humans as a therapeutic strategy for PH. Finally, SH045 (larixyl N-methylcarbamate) represents one of the most selective TRPC6 inhibitors developed to date. Evidence from in vitro and in vivo murine models indicate minimal cross-reactivity with TRPC3 and TRPC7. However, this molecule remains confined to preclinical stages and its clinical applicability has yet to be explored [125,126,131].

TRPC4, implicated in endothelial dysfunction, has also been explored as potential targets in hypoxia-induced PH. Evidence from preclinical models identified ML204 as TRPC4 antagonist, and subsequent studies expanded its evaluation in vivo in models of metabolic dysfunction and migraine [132,133,134]. However, because ML204 can also affect TRPC5, its viability as therapeutic candidate remains limited. Although robust preclinical efficacy has been demonstrated, the absence of human studies precludes definitive conclusions regarding its clinical utility. Moreover, their partial isoform selectivity of current TRPC4 inhibitors raises concerns about redundancy and compensatory responses within TRPC channel family.

In addition to the use of direct TRPC antagonist, an alternative pharmacological approach involves the indirect modulation of TRPC signaling. Huang et al. (2022) [135] investigated the therapeutic effects of sildenafil, a phosphodiesterase-5 (PDE5) inhibitor in neonatal rats exposed to hypoxia and in human (PASMCs). They demonstrated that Sildenafil attenuated right ventricular hypertrophy, elevated mean pulmonary artery pressure, thickening of the distal pulmonary arterial wall and PASMCs proliferation induced by hypoxia. The proposed mechanism is indirect and involves the downregulation of TRPC1 and TRPC6 expression through the upregulation of peroxisome proliferator-activated receptor gamma (PPARγ) [135,136]. Sildenafil is already an FDA-approved drug, initially authorized for erectile dysfunction and later approved for the treatment of PH [137,138]. Nevertheless, due to its non-selective mechanism of action, Sildenafil is associated with adverse effects that limit its long-term therapeutic use [139].

In summary, TRPC6 inhibitors stand out as promising pharmacological candidates for mitigating hypoxia-driven pulmonary vascular remodeling; however, challenges related to isoform selectivity, pharmacokinetic properties, and lack of clinical evaluation have limited their translational progress. Within the context of PH associated with hypoxia and OSA, TRPC6 antagonism remains an attractive yet unproven therapeutic strategy, underscoring the urgent need for translational studies to bridge this gap. Advancing research on TRPC channels is essential as they represent central mediators of disease progression in hypoxia-induced PH and hold significant potential as pharmacological targets for next-generation therapies.

Despite encouraging preclinical data, the lack of isoform-selective TRPC modulators and their limited pharmacokinetic profiles pose major challenges to clinical translation. Improved molecular specificity and high-throughput screening of TRPC6-focused compounds represent necessary steps toward druggability validation.

**Table 3:** Summary of preclinical and clinical studies evaluating TRPC channel modulators in pulmonary hypertension. The table presents the main molecules investigated as TRPC modulators, their primary molecular targets, experimental models, and major outcomes. Abbreviations: CH, chronic hypoxia; PH, pulmonary hypertension; PAH, pulmonary arterial hypertension; PPARγ, peroxisome proliferator-activated-gamma, HPV, hypoxic pulmonary vasoconstriction.

Molecule	Effect	Assay	Study Phase	Main Findings	Limitations	Ref.
BI-749327	TRPC6 inhibition	Whole-cell patch-clamp; Ca^2+^ imaging	Preclinical. In vivo mouse model of chronic hypoxia-induced PH. Human PASMCs	Inhibited acute alveolar hypoxia-induced vasoconstriction. Reverses established PH via regression of pulmonary vascular remodeling.	Not absolutely isoform-selective (~85 selectivity vs. TRPC3)	[129]
SAR7334	TRPC6 inhibition	Whole-cell patch-clamp; Ca^2+^ fluorometry	Preclinical. Isolated perfused mouse lung model for HPV	Abolished hypoxia-induced increases in pulmonary arterial pressure.	Limited selectivity, inhibits TRPC3 and TRPC7 although with lower efficiency	[130]
SH045	TRPC6 inhibition	Patch-clamp recordings; High-throughput Ca^2+^ assays (FLIPR)	Preclinical. Renal fibrosis mouse model.	Attenuate expression of inflammatory and fibrotic markers in renal fibrosis	Limited selectivity, inhibits TRPC3 and TRPC7 although with lower efficiency	[131,140]
ML204	TRPC4 inhibition	Whole-cell patch-clamp; High-throughput Ca^2+^ assays (FLIPR)	Preclinical. Chronic migraine mouse model.	Inhibited visceral pain.	Limited selectivity, inhibits TRPC5 although with lower efficiency	[132,133]
Sildenafil	Indirect down-regulation of TRPC1/TRPC6 via PPARγ signaling	Western blot/qPCR	Clinical. FDA-approved as vasodilator for patients with PAH	Improves exercise capacity and hemodynamics in patients with PAH; Reverses the increase in the right ventricular mean pressure; Attenuate pulmonary arterial remodeling.	Indirect action; adverse effects.	[135,137,141]

## 7. Conclusions and Future Research

Accumulating evidence identifies TRPC channels as important mediators of hypoxia-driven pulmonary hypertension (PH), integrating calcium-dependent signaling, smooth muscle proliferation, endothelial dysfunction, and pulmonary vascular remodeling. Among them, TRPC6 has emerged as the most advanced pharmacological target, as its inhibition consistently attenuates pulmonary vasoconstriction and remodeling in preclinical models. Conversely, while TRPC1, TRPC3 and TRPC4 have been mechanistically implicated in similar pathogenic pathways, the absence of isoform-selective modulators continues to limit their translational exploration.

Future research should focus on developing and validating isoform-specific inhibitors to delineate the precise pathogenic contributions of individual TRPC subtypes. In parallel, advancing current TRPC6 inhibitors into early-phase clinical trials will be essential to determine their safety and therapeutic efficacy in humans. Integrated approaches combining genetic models, selective pharmacology, and comprehensive molecular analyses are needed to clarify compensatory interactions among TRPC isoforms that currently obscure mechanistic understanding.

Taken together, TRPC channels represent a mechanistically coherent and therapeutically compelling framework for addressing both the proliferative and vasoconstrictive components of PH. Although translational to clinical applications remain unrealized, sustained research of TRPC-mediated pathways holds the potential to transform therapeutic strategies for hypoxia- and OSA-associated PH by targeting fundamental molecular drivers of disease progression.

## Figures and Tables

**Table 2:** Expression and functional role of TRPC channels in PAECs and PASMCs. This table summarizes the expression patterns and major functional contributions of TRPC1, TRPC3, TRPC4 and TRPC6 channels in pulmonary vascular cells. Abbreviations: PAECs, pulmonary arterial endothelial cells; PASMCs, pulmonary arterial smooth muscle cells; eNOS, endothelial NO synthase; NO, nitric oxide, CIH, chronic intermittent hypoxia.

TRPC Channel	Expression	Functional Role	Channel Expression
TRPC1	PAECs, PASMCs	Promote vasodilation through activation of eNOS and NO release [8,74,75,76]. Facilitates human PASMCs proliferation and migration [39].	Overexpressed in rat PASMCs exposed to CIH [39]
TRPC3	PAECs, PASMCs	Mediates hyperpolarization-induced vasodilation [77]. Promote human PASMCs proliferation [39]. In human PASMCs promote vasoconstriction [78].	Upregulated in human PASMCs from patients with PAH [39,84]
TRPC4	PAECs, PASMCs	Contributes to vasodilation via eNOS and NO release [8,74,75,76]. Promote human PASMCs proliferation and migration [39].	Overexpressed in rat PASMCs exposed to CIH [39]
TRPC6	PAECs, PASMCs	Stimulate human PASMCs proliferation [39].	Overexpressed in human PASMCs from PAH [39]

## Data Availability

No new data were created or analyzed in this study. Data sharing is not applicable to this article.

## Abbreviations

The following abbreviations are used in this manuscript:

PH	Pulmonary hypertension
PAH	Pulmonary arterial hypertension
IPAH	Idiopathic pulmonary arterial hypertension
HPV	Hypoxic pulmonary vasoconstriction
CH	Chronic hypoxia
CIH	Chronic intermittent hypoxia
OSA	Obstructive sleep apnea
TRPC	Transient receptor potential canonical channel
PASMCs	Pulmonary arterial smooth muscle cells
PAECs	Pulmonary arterial endothelial cells
mPAP	Mean pulmonary pressure
SOCCs	Store-operated calcium channels
ROCCs	Receptor-operated calcium channels
NO	Nitric oxide
ET	Endothelin
VSMCs	Vascular smooth muscle cells
ECs	Endothelial cells
MLCK	Myosin light chain kinase
MLCP	Myosin light chain phosphatase
VDCC	Voltage-dependent calcium channels
STIM	Stromal interaction molecule
SOCE	Store operated current entry
ROCE	Receptor operated current entry
ER	Endoplasmic reticulum
SR	Sarcoplasmic reticulum
CIRB	Calmodulin-and IP3 receptors-binding
RVSP	Right ventricular systolic pressure
RVH	Right ventricular hypertrophy
PLC	Phospholipase C
DAG	Diacylglycerol
PIP2	Phosphatitylinositol 4,5 biphosphate
IP3	Inositol triphosphate
CaM	Ca^2+^-calmodulin
CAMKII	CAM-dependent kinase II
ROS	Reactive oxygen species
eNOS	Endothelial NO synthase
ERK	Extracellular signal regulated kinase
CLQ	Chloroquine
COPD	Chronic obstructive pulmonary disease
CPAP	Continuous positive airway pressure
ICAM	Intercellular adhesion molecule
CRP	C-reactive protein
VCAM	Vascular cell adhesion molecule
NFAT	Nuclear factor of activated T-cells
TNF	Tumor necrosis factor
MMP-9	Matrix metalloproteinase-9
2-APB	Amynoethoxydiphenyl borate
HIF	Hypoxia-inducible factor
AKT	Protein kinase B
mTOR	Mechanistic target of rapamycin
PPAR-γ	Peroxisome proliferator-activated-gamma
